# Validation of a nursing consultation protocol for preterm infants after hospital discharge

**DOI:** 10.1590/0034-7167-2025-0614

**Published:** 2026-10-16

**Authors:** Kelly Saboia Menezes Dias, Alessandra Carvalho Soares Rosa, Wilton Kevin Silva Alencar, Ângela Ferreira Barros

**Affiliations:** I Fundação de Ensino e Pesquisa em Ciências da Saúde, Escola de Saúde Pública do Distrito Federal. Brasília, Distrito Federal, Brazil; II Universidade do Distrito Federal Professor Jorge Amaury Maia Nunes. Brasília, Distrito Federal, Brazil

**Keywords:** Infants, Premature, Patient Discharge, Neonatal Nursing, Primary Care Nursing, Validation Study., Recien Nacido Prematuro, Alta del Paciente, Enfermería Neonatal, Enfermería de Atención Primaria, Estudio de Validación.

## Abstract

**Objective:**

To analyze the evidence of content and semantic validity of a nursing consultation protocol aimed at the care of preterm infants after hospital discharge.

**Methods:**

Methodological study. In the content validation stage, nurse experts with experience in neonatology were selected. After the modifications to the protocol suggested by the experts, semantic validation was carried out with the target audience, composed of nurses working in primary health care.

**Results:**

Eleven experts participated in the content validation, in which the protocol received 70.0% full recommendation and 30.0% recommendation with modifications. In the semantic validation, 30 nurses participated, and the global evaluation showed a full recommendation rate of 93.3% and 6.7% recommendation with modifications.

**Conclusions:**

The protocol demonstrated satisfactory validity evidence, indicating that it can be considered an appropriate tool for outpatient follow up services for preterm infants and for primary health care.

## RESUMEN

**Objetivo:**

Analizar la evidencia de validez de contenido y semántica de un protocolo de consulta de enfermería dirigido al cuidado de recién nacidos prematuros después del alta hospitalaria.

**Métodos:**

Estudio metodológico. En la etapa de validación de contenido, se seleccionaron expertos enfermeros con experiencia en neonatología. Tras las modificaciones del protocolo sugeridas por los expertos, se realizó la validación semántica con el público objetivo, compuesto por enfermeros que actúan en la atención primaria de salud.

**Resultados:**

Once expertos participaron en la validación de contenido, en la cual el protocolo obtuvo un 70,0% de recomendación plena y un 30,0% de recomendación con modificaciones. En la validación semántica participaron 30 enfermeros, y en la evaluación global se obtuvo una tasa de recomendación plena del 93,3% y un 6,7% de recomendación con modificaciones.

**Conclusiones:**

El protocolo presentó evidencia de validez satisfactoria, lo que demuestra que puede considerarse una herramienta adecuada para los servicios ambulatorios de seguimiento de prematuros y para la atención primaria de salud.

## INTRODUCTION

Preterm infants are at risk for developmental difficulties, which increases the likelihood of morbidities, and they are more susceptible to infections and other conditions that may lead to lifelong sequelae^(1)^. Therefore, their families need support from health professionals at every stage - from before conception, through the identification of a high risk pregnancy, the birth of the baby, the period of hospitalization in a health facility, and especially when the baby is discharged and care must continue at home^(2)^.

Hospital discharge is a transition process, also understood as a transfer of care, during which the autonomy of family members regarding continuity of treatment and the importance of post discharge follow up^(3)^ in outpatient services, in conjunction with Primary Health Care (PHC), must be reinforced.

However, there is a discontinuity of care due to weaknesses in the integration between hospital services and PHC. As a result, families must establish new bonds with PHC professionals, who often have limited training to address the specific needs of preterm infants and their families^(4)^. Another study also highlights the complexity of managing the flow of information between the Family Health Strategy (FHS) and other health units, the resistance of families to continue follow up in PHC after the baby is discharged from the neonatal unit, and the limited understanding - both among families and health professionals - of the importance of the FHS in this long term follow up process^(5)^.

In this context, nurses in family health teams play a central role in exercising leadership^(6,7)^ to establish and strengthen a solid bond with the family, enabling the provision of health care^(8)^ articulated with the work of other health professionals and services within the health care network. Therefore, it is important to support PHC nurses in organizing nursing consultations for preterm infants, tailored to their specific needs up to two years of age. This underscores the need to develop and validate a protocol that supports nurses in this specialized and exclusive professional activity.

Care protocols aim to facilitate decision making by describing a specific care situation with detailed operational specifications. In doing so, they provide greater safety for the team, reduce variability in actions, support the incorporation of new technologies and the rational use of resources, and enable the monitoring of process and outcome indicators, contributing to the evaluation of the quality and safety of the care provided^(9)^.

Within this scenario, a nursing consultation protocol for preterm infants after hospital discharge was developed. Following its development, the need for validation was identified, given that this process is essential to ensure the safe use of instruments in clinical practice by verifying whether they achieve their intended objectives and adequately and reliably reflect what they propose to measure^(9)^.

## OBJECTIVE

To analyze the evidence of content and semantic validity of a nursing consultation protocol aimed at the care of preterm infants after hospital discharge.

## METHODS

### Ethical aspects

This study was approved by the Research Ethics Committee of the Health Sciences Teaching and Research Foundation and followed Resolution No. 466/2012 of the *Conselho Nacional de Saúde* (National Health Council). All participants confirmed their agreement by signing the Informed Consent Form (ICF).

### Study design, period, and setting

A methodological validation study was conducted following Pasquali’s theoretical framework, which includes theoretical procedures with content validation in the first stage, followed by empirical procedures with the application of the pilot instrument to the target audience^(10)^ in the second stage. Data collection took place between April and June 2024 in the Federal District. The study was guided by the *Revised Standards for Quality Improvement Reporting Excellence* (SQUIRE 2.0)^(11)^.

### Population and inclusion or exclusion criteria

In the first stage of content validation, the experts were selected using the “snowball” technique, in which one participant referred other potential participants from their professional network^(12)^. Nurses with at least five years of experience in neonatology were included, among them those working in follow up outpatient clinics, given that their expertise could contribute more substantially to content validation. Experts who did not respond to the invitation letter or did not complete the protocol evaluation within ten days of receiving it were excluded.

In the second stage, participants were nurses working in the FHS in Primary Health Units (PHUs) in the Northern Health Region of the Federal District Health Department. The selection of this health region and its PHUs was based on logistical convenience. The Northern Health Region comprises the administrative regions of Sobradinho, Sobradinho II, Fercal, and Planaltina. Approximately 345,286 inhabitants live in this region^(13)^, and 32 PHUs are currently in operation.

Nurses who provide care to preterm children up to two years of age were included. Nurses who were on official leave for more than 30 days during the data collection period, as well as those who, for any reason, did not respond to or return the evaluation instrument within ten days, were excluded.

### Study proctocol

Data recruitment and collection were carried out by the lead researcher. In the first stage, the initial contact identified 22 potential experts, all of whom were invited to participate virtually (via email or WhatsApp®) through an invitation letter.

In the second stage, participant recruitment occurred through phone calls, WhatsApp® messages, or in person contact at the PHUs where they worked. Those who agreed were asked to sign the ICF, sent via a Google Forms link. All participants took part virtually. Participants in both stages received the developed protocol and the evaluation instrument, the Appraisal of Guidelines for Research & Evaluation II - AGREE II^(14)^.

The protocol begins by describing the literature review methodology that supported its development. It then presents an introduction, outlines the situational diagnosis of prematurity in Brazil and in the Federal District, and discusses how the nursing consultation is articulated with the work of the multidisciplinary team in providing care to preterm infants and their families.

The development of the protocol is structured according to the stages of the nursing process. In the nursing assessment stage, emphasis is placed on the use of corrected age, the use of growth charts specific to preterm infants, the evaluation of the history since birth, examinations performed during hospitalization, neonatal screenings, and the physical examination. In the subsequent stage, the NANDA I nursing diagnoses are presented, in which preterm infants appear as a risk population, as well as the ICNP® diagnoses constructed and validated for preterm infants^(15)^. The decision to include both terminologies was based on the understanding of the importance of integrating them to guide the identification of the most appropriate nursing diagnoses according to the professionals’ knowledge^(16)^, and to provide a more accurate representation of the phenomena that are the focus of nursing practice^(17)^. In the planning and implementation stages of nursing care, the protocol includes information on the frequency of consultations, encouragement of the Kangaroo Method, promotion of exclusive breastfeeding, supplementation, vaccination, follow up examinations, guidance on warning signs or risk situations, and, finally, early stimulation.

The AGREE II tool consists of 25 items, including two items for overall assessment and 23 items organized into six quality domains. The overall assessment refers to the global quality and the recommendation of the protocol. Domain 1, “scope and purpose,” refers to the overall objective, specific health questions, and the target population (items 1 to 3); Domain 2, “stakeholder involvement,” considers the participation of relevant stakeholders and represents the views of the target audience (items 4 to 6); Domain 3, “rigor of development,” addresses the process of gathering and synthesizing evidence, the methods used to formulate recommendations, and their respective updates (items 7 to 14); Domain 4, “clarity of presentation,” concerns the language, structure, and format (items 15 to 17); Domain 5, “applicability,” relates to potential facilitators and barriers to implementation, resource implications, and strategies to improve application (items 18 to 21); and Domain 6, “editorial independence,” refers to the development of recommendations free from conflicts of interest (items 22 and 23). Each item was evaluated using a Likert scale ranging from one (strongly disagree) to seven (strongly agree)^(14)^. The experts and nurses were able to add comments about the protocol. Based on their responses, several adjustments were made to improve its adequacy.

### Results’ analysis and statistics

For data analysis, the AGREE II instructions were followed. The calculation for each domain was performed by summing all scores of the individual items and scaling the result as a percentage of the maximum possible score for that domain. The calculation of the total percentage for each domain was obtained using the following formula^(14)^:


$$\frac{\text{Score Obtained - Minimum Possible Score}}{\text{Maximum Possible Score - Minimum Possible Score}}$$

The score obtained corresponds to the sum of the scores for each item. The maximum possible score is calculated by multiplying the highest possible rating by the number of items in the domain and by the number of participants. The minimum possible score is calculated by multiplying the lowest possible rating by the number of items in the domain and by the number of participants. The analyses were performed using Microsoft Excel software. According to AGREE II, there is no minimum required agreement rate for a domain; however, it is up to the user to classify the results and carry out the necessary interventions^(14)^.

## RESULTS

A total of 11 experts participated in the first stage, including three with doctoral degrees, four with master’s degrees, three specialists, and one bachelor prepared nurse with extensive experience in neonatal nursing. All participants had more than 10 years of professional experience. In analyzing the responses, the results were organized by domain, highlighting the agreement rate for each (Table 1), as well as the comments that supported the most substantial modifications to the protocol (Chart 1).

**Table 1:** Agreement rate of the experts in the content validation by domain. Brasília, Distrito Federal, Brazil, 2024

Domain	Taxa de concordância
1. Scope and Purpose	89.3%
2. Stakeholder Involvement	81.3%
3. Rigor of Development	83.9%
4. Clarity of Presentation	86.3%
5. Applicability	73.8%
6. Editorial Independence	61.3%
Overall Protocol Quality	78.0%
Full Recommendation Rate	70.0%
Recommendation with Modifications Rate	30.0%

**Table 2:** Agreement rate of primary care nurses by domain. Brasília, Distrito Federal, Brazil, 2024

Domínio	Taxa de concordância
1. Scope and Purpose	96.6%
2. Stakeholder Involvement	93.8%
3. Rigor of Development	93.8%
4. Clarity of Presentation	95.5%
5. Applicability	92.2%
6. Editorial Independence	91.6%
Overall Protocol Quality	94.2%
Full Recommendation Rate	93.3%
Recommendation with Modifications Rate	6.7%

The comments and suggestions from the experts were carefully analyzed by the researchers. All of them were fully accepted and led to important modifications and adjustments to the protocol, as listed in Chart 1. There was no second round of validation with the experts. Subsequently, the second stage began, in which 30 nurses responded to the form, including one with a doctoral degree, one with a master’s degree, 26 specialists, and two with bachelor’s degrees. Regarding years since graduation, 80% had more than 10 years of experience.

Table 2 presents the percentage values for each domain regarding the evaluation conducted by the nurses, and Chart 2 describes the relevant comments concerning the overall evaluation of the protocol. All agreement rates for each domain were higher in the second stage.

**Chart 1:** Comments or suggestions from the experts in the content validation. Brasília, Distrito Federal, Brazil, 2024

Comments or suggestions	Accepted?
“Since the protocol will be applied up to 2 years of age, the introduction could perhaps address the need for follow-up of this population and not only the third stage of the Kangaroo Method. I also think it would be prudent to verify whether the target population of the protocol is composed of the nurses who care for preterm infants (primary or specialized care?) and not necessarily the preterm infants themselves.” (Participant 03)	Yes
“I would add family members, the mother-baby dyad, father/mother.” (Participant 07)	Yes
“As for the target users of the protocol, I understand that they are the nurses, but I cannot identify which level of care they belong to.” (Participant 03)	Yes
“The protocol mentions the work of other health professionals and the multidisciplinary team, but it does not specify who they are (speech therapists? Physical therapists?).” (Participant 02)	Yes
“I believe the main emphasis for professionals should be on paying attention to risks.” (Participant 05)	Yes
“Gastroesophageal reflux appears as a separate item, but it could be a subitem under possible complications encountered in the first trimester after discharge, or something similar.” (Participant 03)	Yes.
“It is overloaded with information that sometimes applies to the PHC setting and sometimes to the hospital setting; it needs a flowchart to organize the entire process.” (Participant 10)	Yes
“There could be more flowcharts. Perhaps as appendices. They are more visual.” (Participant 09)	Yes
“Tools such as the Bristol and Denver scales could be described as forms of assessment.” (Participant 03)	Yes
“The protocol does not mention the difficulties in applying it.” (Participant 02)	Yes
“I did not identify any suggested indicator for monitoring the implementation of this protocol.” (Participant 06)	Yes
“The section on monitoring by the manager is very superficial.” (Participant 10)	Yes
“There is no statement regarding conflict of interest.” (Participant 05)	Yes
“It will serve as guidance for professionals who care for this group.” (Participant 05)	Yes
“In the situational or clinical diagnosis section, I would include how many preterm infants are born in Brasília and the national and local morbidity and mortality rates; in the birth history section, I would include medical diagnoses from hospitalization and a space to add reasons for possible visits to health services after discharge; I would also describe the physical examination to be performed by the nurse, as well as the reflexes that should be assessed according to the corrected age of the preterm infant.” (Participant 03)	Yes

**Chart 2:** Comments from the overall evaluation of the protocol by primary care nurses. Brasília, Distrito Federal, Brazil, 2024

Comments or suggestions	Accepted?
“Given the difficulties encountered, the solutions were optimized to the fullest.” (Participant 26)	Yes
“Some recommendations regarding the frequency of consultations in Primary Care (weekly and monthly), or the nutritionist participating in 6-month follow-up consultations, for example, do not reflect the current reality of Primary Care, where we attend far more walk-in (emergency) demands than actual prevention and health promotion, which should be the priority. There is also no team training to care for preterm infants.” (Participant 19)	Yes
“Excellent!” (Participant 12)	Yes
“Great protocol! I will base much of the information in my consultations on it.” (Participant 28)	Yes

## DISCUSSION

The protocol demonstrated evidence of satisfactory content and semantic validity, meeting the objective of the study. Thus, it can be considered a tool to support nurses in conducting nursing consultations for preterm infants and their families after hospital discharge, addressing their specific needs, fostering bonding, and enabling self care.

Families of preterm infants face major challenges after hospital discharge due to the discontinuity of care, marked by difficulties in accessing specialized services and by the lack of communication and coordination among different health services. These issues directly affect the health of the children and place a significant burden on families^(4)^. Interest in improving this transition of care after hospital discharge has encouraged the establishment of different forms of in person or remote communication in several countries^(18,19)^, the use of technological tools^(20)^, and the development of various educational technological products for families^(21-23)^ and for health professionals^(3,24-26)^, with the aim of supporting families in providing care to preterm infants and improving the quality of care delivered outside the hospital setting.

In this context, families tend to maintain stronger ties with the hospital services where the infants and their families received neonatal care than with primary care services^(4)^, despite the latter offering greater capillarity and easier access. This reinforces the need to strengthen primary health care (PHC) for the care of preterm infants and their families^(20)^, as well as to expand the knowledge and skills of health professionals to ensure continuous improvement in the quality of care and the promotion of health for preterm newborns^(25)^.

Protocols developed based on scientific evidence and evaluated by experts in the field are valuable and robust tools for clinical practice. The combination of scientific evidence with the practical experience of professionals supports the development of useful tools for daily work. Protocols allow the adaptation of scientific data to the local reality, facilitating their implementation and optimizing outcomes^(8)^.

The validation process conducted with experts led to adjustments in the protocol. Although the AGREE II instrument does not establish a minimum agreement rate^(14)^, the result obtained was below 0.78, the reference value commonly used in content validity index measures^(27)^. This required the acceptance of the suggestions, resulting in substantial changes to the protocol. Subsequently, the results obtained with the target audience helped confirm the improvements made after the adjustments. All validation stages contribute to enhancing the technologies developed and reinforce the assurance of their quality so they can be implemented in health services, guiding more dynamic work processes by supporting practical actions with theoretical foundations^(28)^. It is likely that the improvement in agreement rates in the second validation stage resulted from the modifications made to the protocol based on the experts’ comments.

In Domain 1, “scope and purpose,” higher agreement rates were observed among both experts and primary care nurses. This domain encompasses the overall objective, specific health issues, and the target population. Based on the experts’ comments in this domain, additional information was included to clarify that the protocol can be used in follow up outpatient clinics and in primary health care, as recommended in the third stage of the Kangaroo Method^(29)^.

Based on the comments made in Domain 1, the protocol was expanded to emphasize the importance of identifying the family’s needs and supporting them in caring for the preterm infant. When arriving home with a preterm baby, mothers often experience insecurities and fears. Even for a well trained and actively involved mother, challenges persist once she is at home and must make decisions on her own. Therefore, adequate, frequent, and regular support from health professionals helps to alleviate and prevent the family’s distress^(30,31)^.

In Domain 2, “Stakeholder Involvement,” one of the experts noted the lack of specification regarding which professionals make up the multidisciplinary team. This information was added to the protocol, reinforcing that the coordinated work of the entire team - combining strengths and knowledge - benefits both the infants and their families^(32)^. Follow up care after hospital discharge should be structured with multidisciplinary support across all services within the child health care network^(29)^.

Regarding Domain 3, “Rigor of Development,” the responses and comments did not result in modifications to the protocol. In Domain 4, “Clarity of Presentation,” one expert suggested that gastroesophageal reflux should be emphasized as one of the possible complications. This suggestion aligns with scientific evidence^(33)^. Thus, to improve content presentation, gastroesophageal reflux was added alongside other common problems.

Regarding Domain 5, “Applicability,” one expert suggested the need to describe two tools established by the Ministry of Health. The first is used to assess the neuromotor development of newborns, known as the Denver II, a screening test aimed at identifying children with suspected developmental delay^(34)^, which has been used and recommended in a systematic review^(35)^. Instead of this tool, the protocol opted to use the developmental milestones table from the Child Health Handbook, as health professionals are more familiar with it. The second tool is the Bristol Tongue tie Assessment Protocol, a practical form that can be used by professionals who are not specialists in oral dysfunctions^(36)^. This tool was included in the protocol.

Domain 6, “Editorial Independence,” showed the lowest agreement rate because the experts did not identify a description of conflicts of interest in the protocol. As a result, this information was added and clarified. Presenting conflicts of interest more directly and in greater detail provides greater transparency and credibility to the protocol^(37)^.

With these adjustments, the recommendation rates were satisfactory after both validation stages, suggesting that the protocol presents solid content, structure, and organization, achieving the intended outcome. The systematization of a protocol helps reduce vulnerabilities in the care provided to users and supports the implementation of actions and strategies aimed at ensuring continuity of care, as well as potential modifications to the material when necessary^(4)^.

The use of AGREE II likely contributed to improving the protocol’s consistency and quality, making it an applicable tool with useful implications for practice and generating benefits for both health professionals and patients and their families. Other studies in nursing^(38)^ and health care^(39)^ have also used this tool to evaluate protocols. When the protocol is used alongside other technologies - such as professional training and improvements in care pathways - it may support more comprehensive care for preterm infants, reducing inadequate interventions, hospitalizations, complications, and mortality, while also offering families tools that help reduce anxiety related to caring for a preterm baby^(40)^.

As implications for practice, this protocol equips nurses to conduct nursing consultations for preterm infants after hospital discharge. The nurse’s role with families of preterm babies promotes communication supported by clear technical information and sensitive listening, enabling the construction of a partnership grounded in constructive and collaborative dialogue, which strengthens bonding. This supports the transition of care for a preterm infant into the home environment and helps caregivers adapt - especially the mother, who needs support to reassess her roles and skills in light of her experiences and cultural values^(41)^.

The nursing consultation for preterm infants can be considered an advanced nursing practice due to the complexity and specificity required. It can be incorporated into the range of actions performed in PHC by nurses, who already carry out advanced nursing practices, as evidenced in a national study^(42)^. This reinforces that expanding the scope of advanced nursing practice also requires investment in nurse training and qualification^(43)^.

The next important step would be to monitor the implementation of the protocol during nursing consultations in primary care and outpatient follow up, ensuring its applicability and developmental rigor by making possible adaptations and/or updates according to the specificities of each service.

### Limitations of the study

It is important to highlight that the protocol evaluated by the participants may have appeared extensive and tiring for health professionals to read, as well as the instrument itself that needed to be completed. The online application of the AGREE II may have led to omissions in the professionals’ responses and prevented clarification of doubts regarding the items presented.

### Contributions to the area

The use of technologies with demonstrated validity, such as the present protocol, in nursing practice has the potential to become a supportive tool for conducting consultations with preterm infants across different levels of health care. This contributes to strengthening and expanding nursing competencies.

## CONCLUSION

The study achieved its objective by demonstrating satisfactory evidence of validity and semantic adequacy for the nursing consultation protocol for preterm infants. The evaluation conducted using AGREE II ensured greater rigor and consistency in the analysis of the protocol, thereby supporting the necessary modifications and improvements and providing greater safety for those who will use it. Thus, the protocol can be considered an appropriate tool for application in primary health care and may also be adapted for use in outpatient follow up services for preterm infants or in other settings.

## Data Availability

The research data available in the repository https://repositoriobce.fepecs.edu.br/handle/123456789/1419
