# Supplementary material for: Validation of a nursing consultation protocol for preterm infants after hospital discharge
Source: Rev Bras Enferm. 2026 Oct 16;79:e20250614. doi: 10.1590/0034-7167-2025-0614 (PMC13679167; doi:10.1590/0034-7167-2025-0614)
Supplement: Translation

# Translation: Validação de protocolo de consulta de enfermagem para prematuros após a alta hospitalar

**DOI:** 10.1590/0034-7167-2025-0614pt

**Authors:** Kelly Saboia Menezes Dias, Alessandra Carvalho Soares Rosa, Wilton Kevin Silva Alencar, Ângela Ferreira Barros

**Affiliations:** I Fundação de Ensino e Pesquisa em Ciências da Saúde, Escola de Saúde Pública do Distrito Federal. Brasília, Distrito Federal, Brasil; II Universidade do Distrito Federal Professor Jorge Amaury Maia Nunes. Brasília, Distrito Federal, Brasil

**Keywords:** Recém-Nascido Prematuro, Alta do Paciente, Enfermagem Neonatal, Enfermagem de Atenção Primária, Estudo de Validação.

## Abstract

**Objetivo:**

Analisar as evidências de validade de conteúdo e semântica de um protocolo de consulta de enfermagem voltado ao cuidado de prematuros após a alta hospitalar.

**Métodos:**

Estudo metodológico. Na etapa de validação de conteúdo, selecionaram-se experts enfermeiros com experiência em neonatologia. Após as modificações no protocolo sugeridas pelos experts, foi realizada a validação semântica com o público-alvo composto por enfermeiros atuantes na atenção primária à saúde.

**Resultados:**

Onze experts participaram da validação de conteúdo, na qual o protocolo obteve 70,0% de recomendação plena e 30,0% de recomendação com modificações. Na validação semântica, participaram 30 enfermeiros e obteve-se, na avaliação global, uma taxa de recomendação plena de 93,3% e 6,7% de recomendação com modificações. C**onclusões:** O protocolo apresentou evidências de validade satisfatórias, demonstrando que pode ser considerado como uma ferramenta adequada para serviços ambulatoriais de seguimento de prematuros e na atenção primária à saúde.

## INTRODUÇÃO

Os bebês prematuros apresentam riscos de dificuldades no desenvolvimento, o que acarreta aumento do risco de morbidades, e são mais suscetíveis às infecções e outras enfermidades que podem trazer sequelas para toda a vida^(1)^. Por isso, suas famílias precisam de apoio dos profissionais de saúde em todas as fases, desde antes da concepção, na identificação de uma gravidez de alto risco, no nascimento do bebê, durante sua internação em uma unidade de saúde e, especialmente, quando o bebê recebe alta hospitalar, devendo continuar os cuidados no domicílio^(2)^.

Essa alta é um processo de transição, também entendido como a transferência de cuidado, onde deverá ser reforçada a autonomia dos familiares acerca da continuidade do tratamento e da importância do seguimento pós alta hospitalar^(3)^ no serviço ambulatorial, em conjunto com a Atenção Primária à Saúde (APS).

Entretanto, ocorre uma descontinuidade do cuidado devida às fragilidades da integração do serviço hospitalar com a APS, sendo necessário que a família estabeleça novos vínculos com os profissionais da APS que possuem uma pouca qualificação para atender às peculiaridades dos prematuros e suas famílias^(4)^. Outro estudo destaca ainda a complexidade em gerenciar o fluxo de informações entre a Estratégia Saúde da Família (ESF) e demais unidades de saúde, a resistência das famílias em dar prosseguimento ao acompanhamento na APS após a alta do bebê da unidade neonatal, além da percepção limitada, tanto por parte das famílias quanto dos profissionais de saúde, sobre a importância da ESF nesse processo de acompanhamento a longo prazo^(5)^.

Nesse contexto, os enfermeiros das equipes de saúde da família têm um papel central no exercício da liderança^(6,7)^ para estabelecer e fortalecer sólido vínculo com a família para proporcionar o cuidado em saúde^(8)^ articulado com a atuação dos outros profissionais de saúde e outros serviços da rede de atenção à saúde. Dessa forma, é importante apoiar os enfermeiros da APS para organizarem as ações das consultas de enfermagem de prematuros direcionadas para suas especificidades até os dois anos de vida, emergindo assim necessidade de elaboração e validação de um protocolo que apoie os enfermeiros nesta atividade privativa e especializada.

Os protocolos assistenciais objetivam facilitar a tomada de decisão, descrevendo uma situação específica de cuidado com detalhes e especificações operacionais. Dessa maneira, trazem maior segurança à equipe, reduzem a variabilidade das ações, facilitam a incorporação de novas tecnologias e uso racional dos recursos, além de possibilitarem o monitoramento dos indicadores de processo e resultados, contribuindo para a avaliação da qualidade e segurança do cuidado prestado^(9)^.

Diante disso, procedeu-se, portanto, a elaboração de um protocolo de consulta de enfermagem para prematuros após alta hospitalar. Após isso, identificou-se a necessidade de sua validação, tendo em vista que esse processo é indispensável para garantir a segurança no uso de instrumentos na prática clínica, ao verificar se esses alcançam os objetivos propostos e contemplam de forma adequada e fidedigna o que se propõem^(9)^.

## OBJETIVO

Analisar as evidências de validade de conteúdo e semântica de um protocolo de consulta de enfermagem voltado ao cuidado de prematuros após a alta hospitalar.

## MÉTODOS

### Aspectos éticos

Essa pesquisa foi aprovada pelo Comitê de Ética em Pesquisa da Fundação de Ensino e Pesquisa em Ciências da Saúde e seguiu a resolução nº 466/2012 do Conselho Nacional de Saúde. Todos os participantes confirmaram o aceite por meio da assinatura do Termo de Consentimento Livre e Esclarecido (TCLE).

### Desenho, período e local de estudo

Realizou-se um estudo metodológico de validação seguindo o referencial teórico de Pasquali, compreendendo procedimentos teóricos com a validação de conteúdo na primeira etapa e em seguida os empíricos, com a aplicação do instrumento piloto com o público-alvo^(10)^ na segunda etapa. A coleta de dados ocorreu entre abril e junho de 2024, no Distrito Federal. O estudo foi norteado pelo *Revised Standards for Quality Improvement Reporting Excellence* (SQUIRE 2.0)^(11)^.

### População e critérios de inclusão ou exclusão

Na primeira etapa de validação de conteúdo, os experts foram selecionados por meio da técnica da “bola de neve” em que um participante indicava outros possíveis participantes a partir de sua rede de contatos^(12)^. Foram incluídos enfermeiros com experiência de, no mínimo, cinco anos em neonatologia, dentre eles enfermeiros atuantes em ambulatórios de seguimento, tendo em vista que a expertise desses profissionais poderia colaborar mais para a validação do conteúdo. Foram excluídos experts que não responderam a carta convite e avaliação do protocolo em até dez dias após o envio.

Na segunda etapa, os participantes foram enfermeiros atuantes na ESF nas Unidades Básicas de Saúde (UBS) da Região de Saúde Norte da Secretaria de Estado de Saúde do Distrito Federal. A seleção dessa região de saúde e UBS ocorreu por conveniência logística. A Região de Saúde Norte corresponde às regiões administrativas de Sobradinho, Sobradinho II, Fercal e Planaltina. Nesta região de saúde, residem,aproximadamente, 345.286 habitantes^(13)^ e estão em funcionamento 32 UBS.

Foram incluídos enfermeiros que atendem crianças prematuras até dois anos de vida. Foram excluídos os enfermeiros que estavam em afastamento oficial superior a 30 dias durante o período da coleta de dados e aqueles que, por qualquer motivo, não responderam ou não entregaram o instrumento de avaliação em até dez dias.

### Protocolo de estudo

Os recrutamentos e coleta de dados foram realizados pela pesquisadora responsável. Na primeira etapa, o contato inicial permitiu elencar um quantitativo de 22 possíveis experts, e todos foram convidados para participação de forma virtual (e-mail ou *Whatsapp®*) com o envio da carta convite.

Na segunda etapa, o recrutamento dos participantes ocorreu por meio de contato telefônico, mensagem de *Whatsapp®* ou presencialmente na UBS na qual eles atuam. Aos que aceitaram, foi solicitada a assinatura do TCLE enviado por link do *Google Forms.* Todos participaram de forma virtual. Os participantes das duas etapas receberam o protocolo elaborado e o instrumento para avaliação, denominado *Appraisal of Guidelines for Research & Evaluation II* - AGREE II^(14)^.

O protocolo inicia descrevendo a metodologia de revisão da literatura que fundamentou sua elaboração. Em seguida, apresenta uma introdução, descreve o diagnóstico situacional da prematuridade no Brasil e no Distrito Federal, e a articulação da consulta de enfermagem com a atuação da equipe multiprofissional para assistência ao prematuro e sua família.

O desenvolvimento do protocolo se estrutura conforme as etapas do processo de enfermagem. Na etapa avaliação de enfermagem, enfatizam-se o uso da idade corrigida, uso das curvas de crescimento específicas para bebês pré-termo, avaliação do histórico desde o nascimento, exames realizados na internação, triagens neonatais e exame físico. Na etapa subsequente, apresentam-se os diagnósticos de enfermagem da NANDA-I, em que os prematuros aparecem como populações de risco, e os da CIPE®, construídos e validados para prematuros^(15)^. Optou-se por incluir as duas terminologias por compreender a importância da integração delas para nortearem a identificação dos diagnósticos de enfermagem mais adequados conforme o conhecimento dos profissionais^(16)^, e uma representação mais acurada dos fenômenos que são objetos da prática da enfermagem^(17)^. Nas etapas de planejamento e implementação de enfermagem constam informações sobre a periodicidade das consultas, incentivo ao uso do Método Canguru, encorajamento ao aleitamento materno exclusivo, suplementação, vacinação, exames de controle, orientações para sinais de alerta ou situações de risco e, por fim, estimulação precoce.

O instrumento AGREE II é composto por 25 itens, sendo dois itens de avaliação global e 23 itens organizados em seis domínios de qualidade. A avaliação global refere-se à qualidade global e à recomendação do protocolo. O domínio 1 compreende “escopo e finalidade”e refere-se ao objetivo geral, às questões específicas de saúde e à população-alvo (itens 1 a 3); domínio 2, “envolvimento das partes interessadas”, considera a participação das partes interessadas e representa a visão do público alvo (itens 4 a 6); domínio 3, “rigor do desenvolvimento”, trata sobre o processo para coletar e sintetizar as evidências, os métodos para a formulação das recomendações e a suas respectivas atualizações (itens 4 a 6); domínio 4, “clareza da apresentação”, aborda a linguagem, estrutura e o formato (itens 15 a 17), domínio 5, “aplicabilidade”, relaciona-se aos prováveis facilitadores e barreiras para implementação, envolvimento de recursos e estratégias para melhorar a aplicação (itens 18 a 21); domínio 6, “independência editorial”, refere-se à elaboração de recomendações sem conflito de interesses (itens 22 e 23). Cada item foi avaliado por uma escala do tipo Likert com pontuação de um (discordo totalmente) a sete (concordo totalmente)^(14)^. Os experts e os enfermeiros puderam acrescentar comentários sobre o protocolo. A partir das respostas, foram realizados vários ajustes para melhor adequá-lo.

### Análise dos resultados e estatística

Para análise dos dados, seguiram-se as instruções do próprio AGREE II. O cálculo para cada domínio foi realizado pela soma de todas as pontuações dos itens individuais e escalonado como porcentagem da pontuação máxima possível no domínio. O cálculo da porcentagem total do domínio foi obtido seguindo a fórmula^(14)^:


$$\frac{\text{Pontuação obtida - Pontuação mínima possível}}{\text{Pontuação máxima possível - Pontuação mínima possível}}$$

A pontuação obtida é a soma das pontuações de cada item. A pontuação máxima possível é a multiplicação da maior nota possível pela quantidade de itens do domínio multiplicada pelo número de participantes. A pontuação mínima possível é a multiplicação da menor nota possível pela quantidade de itens do domínio multiplicada pelo número de participantes. As análises foram realizadas utilizando o software Microsoft Excel. De acordo com o AGREE II, não existe taxa de concordância mínima em um domínio, mas cabe ao usuário classificar e realizar as intervenções necessárias^(14)^.

## RESULTADOS

Participaram da primeira etapa 11 experts, dentre eles três doutores, quatro mestres, três especialistas e um graduado com vasta experiência em enfermagem neonatal. Em relação ao tempo de formação, todos os participantes possuíam mais de 10 anos. Na análise das respostas, realizou-se a separação por domínio destacando a taxa de concordância de cada um (Tabela 1), bem como os comentários que subsidiaram as maiores modificações no protocolo (Quadro 1).

**Tabela 1:** Taxa de concordância dos experts da validação de conteúdo por domínio. Brasília, Distrito Federal, Brasil, 2024

Domínio	Taxa de concordância
1. Escopo e finalidade	89,3%
2. Envolvimento das partes	81,3%
3. Rigor do desenvolvimento	83,9%
4. Clareza da apresentação	86,3%
5. Aplicabilidade	73,8%
6. Independência editorial	61,3%
Qualidade global do protocolo	78,0%
Taxa de recomendação plena	70,0%
Taxa de recomendação com modificações	30,0%

**Quadro 1:** Comentários ou sugestões dos experts da validação de conteúdo. Brasília, Distrito Federal, Brasil, 2024

Comentários ou sugestões	Aceita ou não
“Como o protocolo será aplicado até 2 anos de idade, a introdução talvez pudesse abarcar sobre a necessidade de seguimento desta população e não apenas sobre a terceira etapa do canguru. Acho prudente também verificar se a população-alvo do protocolo não é constituída pelos enfermeiros que atendem os prematuros (primária ou especializada?) e não necessariamente pelos prematuros”. (Participante 03)	Aceita.
“Acrescentaria: familiares, binômio, pai/mãe”. (Participante 07)	Aceita.
“Como usuários-alvo do protocolo, entendo que são os enfermeiros, mas não identifico de qual nível de atenção”. (Participante 03)	Aceita.
“No protocolo cita a atuação de outros profissionais de saúde e da equipe multidisciplinar, mas não especifica quais são eles (fonoaudiólogos? Fisioterapeutas?)”. (Participante 02)	Aceita.
“Acredito que a ênfase maior, para os profissionais, é atentar aos riscos”. (Participante 05)	Aceita
“O refluxo gastroesofágico aparece como item separado, podendo ser um subitem de possíveis intercorrências encontradas no primeiro trimestre pós-alta ou algo assim”. (Participante 03)	Aceita.
“está carregado de informações que ora se aplica no cenário da APS ora da instituição hospitalar, precisa de um fluxograma para organizar todo esse processo”. (Participante 10)	Aceita.
“Poderia ter mais fluxogramas. Talvez como anexos. São mais visuais”. (Participante 09)	Aceita.
“Ferramentas como o Bristol e o Denver poderiam ser descritos como formas de avaliações”. (Participante 03)	Aceita.
“No protocolo não há menção sobre as dificuldades para aplicação do mesmo” (Participante 02)	Aceita.
“Não identifiquei a sugestão de indicador para monitoramento da implantação para este protocolo”. (Participante 06)	Aceita.
“muito superficial, ao mencionar o monitoramento feito pelo gestor”. (Participante 10)	Aceita.
“Não há registro do conflito de interesses”. (Participante 05)	Aceita.
“Será norteador ao profissional que atende este grupo”. (Participante 05)	Aceita.
“Diagnóstico situacional ou clínico colocaria quantos prematuros nascem em Brasília e as taxas de morbimortalidade nacionais e locais; no histórico de nascimento até o momento, colocar diagnósticos médicos de internação e um espaço para acrescentar razões de possíveis visitas aos serviços de saúde após a alta; descrever o exame físico que será realizado pelo enfermeiro, assim como demais reflexos que deverão ser avaliados de acordo com a idade corrigida do prematuro”. (Participante 03)	Aceita.

Os comentários e sugestões dos experts foram analisados detalhadamente pelos pesquisadores. Todos foram aceitos integralmente e geraram importantes modificações e ajustes no protocolo, conforme elencado no Quadro 1. Não houve segunda rodada de validação com os experts. Em seguida, iniciou-se a segunda etapa, na qual responderam ao formulário 30 enfermeiros, entre eles um doutor, um mestre, 26 especialistas e dois graduados. Quanto ao tempo de formação, 80% possuem mais de 10 anos. Em relação ao tempo de experiência em atenção primária, 46,7% dos participantes possuem mais de 10 anos.

A Tabela 2 apresenta os valores das porcentagens de cada domínio referente a avaliação realizada pelos enfermeiros e o Quadro 2 descreve os comentários relevantes referentes a avaliação global do protocolo. Todas as taxas de concordância de cada domínio foram maiores na segunda etapa.

**Quadro 2:** Comentários da avaliação global do protocolo pelos enfermeiros da atenção primária. Brasília, Distrito Federal, Brasil, 2024

Comentários ou sugestões	Aceita ou não
“Frente às dificuldades encontradas as soluções foram otimizadas ao máximo” (Participante 26)	Aceita.
“Algumas orientações sobre frequência de consultas na Atenção Básica (semanal e mensal) ou nutricionista participar de consultas de CD de 6 meses, por exemplo, não consideram a realidade da Atenção Básica nos dias de hoje, onde atendemos muito mais demandas espontâneas (emergências) do que propriamente prevenção e promoção à saúde, o que deveria ser a prioridade. Também não há capacitação da equipe para atender prematuros”. (Participante 19)	Aceita.
“Excelente!” (Participante 12)	Aceita.
“Ótimo protocolo! Vou basear muita informação dele nas minhas consultas” (Participante 28)	Aceita.

**Tabela 2:** Taxa de concordância dos enfermeiros da atenção primária por domínio. Brasília, Distrito Federal, Brasil, 2024

Domínio	Taxa de concordância
1. Escopo e finalidade	96,6%
2. Envolvimento das partes	93,8%
3. Rigor do desenvolvimento	93,8%
4. Clareza da apresentação	95,5%
5. Aplicabilidade	92,2%
6. Independência editorial	91,6%
Qualidade global do protocolo	94,2%
Taxa de recomendação plena	93,3%
Taxa de recomendação com modificações	6,7%

## DISCUSSÃO

O protocolo apresentou evidências de validade de conteúdo e semântica satisfatória, atendendo ao objetivo do estudo. Dessa forma, ele pode ser considerado como uma ferramenta para ajudar os enfermeiros a realizarem a consulta de enfermagem dos prematuros e suas famílias após a alta hospitalar, atendendo às suas especificidades, constituindo vínculo e capacitando para autocuidado.

As famílias de bebês prematuros enfrentam grandes desafios após a alta hospitalar com a descontinuidade do cuidado, marcada pela dificuldade de acesso a serviços especializados e pela falta de comunicação e coordenação entre os diferentes serviços de saúde, o que impacta diretamente na saúde das crianças e sobrecarrega as famílias^(4)^. O interesse em melhorar essa transição do cuidado após a alta hospitalar tem instigado o estabelecimento de diferentes formas de comunicação presencial ou remota em diversos países^(18,19)^, o uso de ferramenta tecnológica^(20)^e o desenvolvimento de diferentes produtos tecnológicos educacionais para familiares^(21-23)^ e para profissionais de saúde^(3,24-26)^, a fim de apoiarem as famílias para fornecerem o cuidado ao prematuro e qualificarem a assistência prestada fora do âmbito hospitalar.

Nesse contexto, o vínculo das famílias permanece sendo maior com os serviços hospitalares nos quais os bebês e suas famílias foram atendidos no período neonatal do que com os serviços de atenção primária^(4)^ que apresentam maior capilaridade e facilidade para acesso. Isso reforça a necessidade de capacitar a APS para o atendimento dos prematuros e suas famílias^(20)^, além de ampliar o conhecimento e as habilidades dos profissionais de saúde visando à melhoria contínua da qualidade dos cuidados e à promoção da saúde dos recém-nascidos prematuros^(25)^.

Protocolos elaborados com base em evidências científicas e avaliados por especialistas na área são ferramentas valiosas e fortes para a prática clínica. Essa união de evidências científicas com a experiência prática dos profissionais ajuda na estruturação de ferramentas úteis para o dia a dia. Os protocolos permitem a adaptação dos dados científicos à realidade local, facilitando sua implementação e otimizando os resultados^(8)^.

O processo de validação feito com experts proporcionou ajustes no protocolo. Apesar de o instrumento AGREE II não considerar uma taxa de concordância mínima^(14)^, o resultado foi inferior à 0,78, valor de referência utilizado em medidas de índice de validade de conteúdo^(27)^. Isso exigiu que as sugestões fossem aceitas, provocando mudanças substanciais no protocolo. Em seguida, o resultado alcançado com o público-alvo corroborou para confirmar as melhorias do protocolo após os ajustes. Todas as etapas de validação favorecem a melhoria das tecnologias desenvolvidas e reforçam a garantia de sua qualidade para que sejam implantadas em serviços de saúde, orientando processos de trabalho mais dinâmicos ao subsidiarem a atuação prática com fundamentação teórica^(28)^. Possivelmente, a melhoria nas taxas de concordância na segunda etapa de validação foi decorrente das modificações feitas no protocolo a partir dos comentários dos experts.

No domínio 1 “escopo e finalidade” verificou-se maior taxa de concordância pelos experts e enfermeiros da atenção primária. Esse domínio abarca o objetivo geral, as questões específicas de saúde e a população alvo. A partir dos comentários dos experts neste domínio, foram inseridas mais informações para esclarecer que o protocolo pode ser utilizado no ambulatório de seguimento e na atenção primária à saúde, conforme preconizado pela terceira etapa do método canguru^(29)^.

Ainda com base nos comentários feitos no domínio 1, foi ampliada no protocolo a relevância de identificar as necessidades da família e apoiá-la para cuidar do prematuro. Ao chegar em casa com um bebê prematuro, as mães se encontram com inseguranças e medos. Mesmo para uma mãe participativa e bem treinada, os desafios permanecem quando está em casa e precisa tomar as decisões sozinha. Portanto, o suporte adequado, frequente e regular dos profissionais de saúde ajuda a amenizar e prevenir os sofrimentos dessa família^(30,31)^.

No domínio 2 “Envolvimento das partes”, um dos experts comentou sobre a falta de especificação de quais profissionais participam da equipe multiprofissional. Essa informação foi incluída no protocolo, reforçando que a atuação de toda a equipe, unindo forças e conhecimentos, beneficia os bebês e suas famílias^(32)^. O acompanhamento de seguimento pós alta hospitalar deve ser estruturado com o atendimento multiprofissional em todos os serviços da rede de atenção à saúde da criança^(29)^.

Quanto ao domínio 3, “Rigor do Desenvolvimento”, as respostas e comentários não resultaram em modificações no protocolo. No domínio 4, “Clareza da Apresentação”, um dos experts sugeriu que o assunto refluxo gastroesofágico fosse enfatizado como uma das intercorrências. Essa sugestão corrobora com as evidências científicas^(33)^. Dessa forma, para melhorar a apresentação do conteúdo, o refluxo gastroesofágico foi adicionado junto aos outros problemas mais comuns.

Em relação ao domínio 5, “Aplicabilidade”, um expert sugeriu a necessidade de descrição de duas ferramentas estabelecidas pelo Ministério da Saúde. A primeira é utilizada para avaliação do desenvolvimento neuromotor do recém-nascido, denominado Denver II, que é um teste de triagem cujo objetivo é identificar crianças com suspeita de atraso no desenvolvimento^(34)^, utilizada e recomendada em estudo de revisão sistemática^(35)^. Ao invés dessa ferramenta, optou-se por utilizar no protocolo a tabela de desenvolvimento da própria caderneta da criança porque os profissionais de saúde estão mais familiarizados com ela. A segunda é um protocolo para o teste da linguinha do modelo de Bristol, que se caracteriza como um formulário prático e pode ser utilizado por profissionais não especialistas em disfunções orais^(36)^. Essa ferramenta foi incluída no protocolo.

O domínio 6, “Independência Editorial”, obteve a menor taxa de concordância porque os experts não identificaram a descrição dos conflitos de interesse no protocolo. A partir disso, estas informações foram incluídas e esclarecidas. Ao demonstrar os conflitos de interesses de forma mais direta e detalhada, confere-se maior transparência e credibilidade ao protocolo^(37)^.

Com isso, as taxas de recomendação foram satisfatórias após as duas etapas de validação, sugerindo que o protocolo apresenta bom conteúdo, estrutura e organização, alcançando o resultado pretendido. A sistematização de um protocolo auxilia na diminuição da vulnerabilidade dos cuidados prestados aos usuários, favorece a implementação de ações e estratégias com objetivo de garantir a continuidade dos cuidados e possíveis modificações ao material, se considerado necessário^(4)^.

O uso do AGREE II provavelmente possibilitou melhorar a consistência e qualidade dele, tornando-o um material aplicável com implicações úteis para a prática e gerando benefícios, tanto para os profissionais de saúde quanto para os pacientes e suas famílias. Outros estudos na área da enfermagem^(38)^ e da saúde^(39)^ também utilizam essa ferramenta para avaliar protocolos. Com a utilização do protocolo agregado a outras tecnologias, dentre elas a capacitação dos profissionais de saúde e melhoria dos fluxos assistenciais, pode-se alcançar um cuidado mais integral ao prematuro, diminuindo as intervenções inadequadas, internações, intercorrências e mortalidade, assim como oferecendo às famílias ferramentas que diminuam a ansiedade relacionada aos cuidados com o bebê prematuro^(40)^.

Como implicações para a prática, este protocolo instrumentaliza os enfermeiros para exercerem e realizarem a consulta de enfermagem de prematuros após a alta hospitalar. A atuação do enfermeiro junto às famílias de bebês prematuros favorece uma interlocução com informações técnicas apresentadas de forma clara, com escuta sensível que permite a construção de uma parceria com diálogo construtivo e colaborativo, o que possibilita formação de vínculos. Isso auxilia a transição do cuidado de um bebê prematuro para o ambiente domiciliar, e ajuda os cuidadores a se adaptarem, principalmente a mãe, que precisa de suporte para reavaliar seus papéis e habilidades à luz de suas experiências e valores culturais^(41)^.

A consulta de enfermagem de bebês prematuros pode ser considerada uma prática avançada de enfermagem devido à complexidade e a especificidade que tal prática exige. Pode ser incluída no repertório de ações executadas na APS por enfermeiros, os quais já exercem as práticas avançadas de enfermagem conforme evidenciado em estudo nacional^(42)^, e reforça que a ampliação do escopo de ações da prática avançada de enfermagem também perpassa investimentos para qualificação dos enfermeiros^(43)^.

O próximo passo importante seria acompanhar a implementação do protocolo nas consultas de enfermagem na atenção primária e no seguimento ambulatorial, buscando manter sua aplicabilidade e seu rigor de desenvolvimento realizando possíveis adaptações e/ou atualizações que podem ser relacionadas às especificidades de cada serviço.

### Limitações do Estudo

Destaca-se que o protocolo avaliado pelos participantes pode ter se demonstrado extenso e cansativo para leitura dos profissionais de saúde, bem como o próprio instrumento a ser respondido. A aplicação do AGREE II de maneira online pode ter gerado omissões quanto às respostas dos profissionais e impossibilitado a retirada de dúvidas quanto aos itens apresentados.

### Contribuições para a Área

O uso de tecnologias com evidências de validade como o presente protocolo na prática da enfermagem tem potencial para tornar-se um instrumento auxiliar na realização dessa consulta em serviços de diferentes níveis de atenção à saúde, potencializando as competências da enfermagem.

## CONCLUSÃO

O estudo alcançou seu objetivo ao verificar evidências de validade e semântica satisfatórias para o protocolo de consulta de enfermagem para prematuros. A avaliação feita por meio do AGREE II garantiu maior rigor e consistência na análise do protocolo, e dessa forma garantiu as modificações e as melhorias necessárias no protocolo, conferindo maior segurança a quem utilizá-lo. Dessa forma, o protocolo pode ser considerado como uma ferramenta adequada para ser aplicado na atenção primária à saúde, podendo também ser adaptado para uso em serviços ambulatoriais de seguimento de prematuros ou em outras localidades.

## Data Availability

Os dados de pesquisa disponíveis em repositório: https://repositoriobce.fepecs.edu.br/handle/123456789/1419
